# Adjuvants are Key Factors for the Development of Future Vaccines: Lessons from the Finlay Adjuvant Platform

**DOI:** 10.3389/fimmu.2013.00407

**Published:** 2013-12-02

**Authors:** Oliver Pérez, Belkis Romeu, Osmir Cabrera, Elizabeth González, Alexander Batista-Duharte, Alexis Labrada, Rocmira Pérez, Laura M. Reyes, Wendy Ramírez, Sergio Sifontes, Nelson Fernández, Miriam Lastre

**Affiliations:** ^1^Immunology Department, Research and Development Vice-presidency, Finlay Institute, Havana, Cuba; ^2^Havana Medical Sciences University, Havana, Cuba; ^3^Toxicology and Biomedicine Center (TOXIMED), Medical Sciences University Santiago de Cuba, Santiago de Cuba, Cuba; ^4^Centro Nacional de Biopreparados (BioCen), Mayabeque, Cuba; ^5^Centro de Bioactivos Químicos (CBQ), Universidad Central Marta Abreu de Las Villas, Villa Clara, Cuba; ^6^School of Biological Sciences, University of Essex, Essex, UK

**Keywords:** adjuvant, vaccine, proteoliposome, cochleate, allergen, fish

## Abstract

The development of effective vaccines against neglected diseases, especially those associated with poverty and social deprivation, is urgently needed. Modern vaccine technologies and a better understanding of the immune response have provided scientists with the tools for rational and safer design of subunit vaccines. Often, however, subunit vaccines do not elicit strong immune responses, highlighting the need to incorporate better adjuvants; this step therefore becomes a key factor for vaccine development. In this review we outline some key features of modern vaccinology that are linked with the development of better adjuvants. In line with the increased desire to obtain novel adjuvants for future vaccines, the Finlay Adjuvant Platform offers a novel approach for the development of new and effective adjuvants. The Finlay Adjuvants (AFs), AFPL (proteoliposome), and AFCo (cochleate), were initially designed for parenteral and mucosal applications, and constitute potent adjuvants for the induction of Th1 responses against several antigens. This review summarizes the status of the Finlay technology in producing promising adjuvants for unsolved-vaccine diseases including mucosal approaches and therapeutic vaccines. Ideas related to adjuvant classification, adjuvant selection, and their possible influence on innate recognition via multiple toll-like receptors are also discussed.

## Introduction

Since the systematization of vaccination principles by Louis Pasteur in 1886 and the introduction of the “triple I” (isolation, inactivation, and injection) concept, most vaccinologists have searched for specific antigens to be used as immunogens, with special emphasis on epitope identification. This approach has also aimed at reducing vaccine reactogenicity and hence increasing efficacy. Since early times (1), adjuvants have been considered a crucial vaccine component. Indeed, adjuvants play a key role in most new vaccine formulations and are used to enhance the efficacy of a particular preparation, even when different vaccines are prepared with identical antigens (2). Currently, several pharmaceutical companies use the aluminum-based mineral salts (Alum) as an adjuvant; which include three different aluminum salts: aluminum hydroxide, aluminum phosphate, and aluminum hydroxyphosphate (3). Alum is found in numerous vaccines, including diphtheria-tetanus-pertussis, human papillomavirus, influenza, and hepatitis vaccines (4), and most importantly, this adjuvant has an excellent health and safety track record (5). Alum provokes responses characterized by a predominance of immunoglobulin G 1 (IgG1) in mice and IgE (in mice and humans). Notably, however, Alum is known to be a poor adjuvant for the induction of cytotoxic T-cell immunity and T helper (Th) 1 immune responses, which are essential to combat several life-threatening infections. Thus, there is an urgent need to develop novel adjuvants to address the development of vaccines against pathogens that have so far been refractory to traditional vaccination strategies and to overcome the limitations of the few available licensed adjuvants (3, 6).

Recent advances in immunology and related disciplines such as genomics and proteomics have contributed enormously to the field of vaccinology and have permitted the rational design of adjuvants and their molecular characterization. An important advance has been the understanding as to how the innate immune system is able to “sense” and recognize molecules associated with specific families of microbes, termed microbe-associated molecular patterns (MAMP). This recognition occurs via a series of pattern-recognition receptors (PRRs), including toll-like receptors (TLRs), lectin-type receptors, and soluble cytoplasmic receptors (Nod-like receptors and retinoic acid inducible gene I-like receptors). The aim of this review is to discuss new ideas about vaccinology and adjuvant technology and to consider how it may be possible to move forward with alternative new potent adjuvants. The article also contains brief summaries of recent experimental data obtained by the Finlay Adjuvant (AF) platform, based on proteoliposomes (PL), PL-derived cochleates (Co), and non PL-derived Co for the development of prophylactic and therapeutic vaccines.

## Key Features in Modern Vaccinology Linked with the Development of Adjuvants

### Immunopolarized adjuvants: Cornerstones to design effective vaccines

An accurate classification of existing adjuvants has been difficult to achieve. This is due to their great diversity; in many cases the mechanism of action is still unknown (7). The most appropriate existing classifications include two main groups. The first is defined as facilitators of signal 1, signal 2, and/or signal 3 (8); these signals are able to induce an immune response (2, 9). In accordance with these functional characteristics, adjuvants can facilitate T-cell receptor (TCR) engagement, antigen capture by dendritic cells (DC) at the inoculation site, and delivery to particular sites of the regional lymph nodes (signal 1) co-stimulation, with the up-regulation of soluble and membrane co-stimulatory signals (signal 2), and an inflammatory stimulus by activating Th cells (signal 3) via cytokines. The second group takes into account the role of adjuvants as immunopotentiators (IP) and/or delivery system (DS) (10). Nevertheless, we consider that the infected host requires a particular kind of immune response to protect itself against each infection. Consequently, we have included immunopolarization (IPz) as a third category, which is totally independent of those defined above (3, 11). With the vast information available today on the role of MAMPs in the induction of immune responses and knowing the IPz properties of an adjuvant, it should be possible to tailor a vaccine to selectively induce the desired response against specific infections. It is now clear that different subsets of helper T-cells, such as Th1, Th2, Th3, Th9, Th17, and follicular Th (Tfh) and T regulatory cells are part of the cell-mediated immune protection against different pathogens. Several adjuvants have been shown to be capable of stimulating more than one type of cell-mediated immune response. This concept has led us to introduce the idea that an IPz category is one that exhibits multiple stimulatory properties (activation of multiple TLRs), in different directions (activation of different T-cells subsets) of the immune response. Notably, adjuvants may share more than one of these properties (IP, DS, and IPz properties) (12). For example, the yellow fever vaccine YF-17D, one of the most effective vaccines available, stimulates innate and adaptive immunity through its ability to activate DCs via TLR-2, TLR-7, TLR-8, and TLR-9 (13). The triggering of multiple and simultaneous TLRs increases the production of cytokines IL-12 and IL-23, leading to synergistic activation of DCs, with enhanced and sustained Th1-polarizing capacity (14). Consistent with the stimulation of multiple facets of the immune response, it is well known that CpG dinucleotides redirect isotype production toward Th1, via TLR-9 and MyD88 (15). Recently, Mastelic et al. (16) have demonstrated that adjuvant/DSs like CpG oligonucleotides markedly increase germinal center Tfh cell and germinal center B cell responses in neonates. In other applications, immunopolarized adjuvant combinations could prove an important strategy in viral protection and cancer therapy, since TLR agonists may prime tumor cells to become targets for cytotoxic agents. In this context, use of a mouse model showed an increased protective efficacy of vaccination with a human immunodeficiency virus (HIV) envelope peptide following combination of three TLR agonists, TLR-2/6, TLR-3, and TLR-9 (17). Furthermore, in cancer immunity, it was observed that autologous tumor cells mixed with bacillus Calmette–Guerin (BCG) were of significant clinical benefit for patients with Stage II colon cancer (18).

### Adjuvants and the stimulation of polarized Th responses

Most immunologists, particularly vaccinologists, consider antibodies the most important immune markers induced by vaccination (19). However, this categorization is not absolute, because if we eliminate T-cells, we certainly neither produce antibodies nor induce any long sustained protection. Often, however, antibody-mediated protection against a pathogen has been considered sufficiently effective, commensurate for vaccines to be licensed. Clearly, antibodies are more easily detected and quantified than cellular responses, indeed they are used to define the correlate of protection of several vaccines, based on antibody concentration, for example *Haemophylus* and pneumococcal vaccines or functional, such as bactericidal or opsonophagocytic in *Neisseria* vaccines. In the late 1980s, the concept that Th1 cells (cellular immunity) conferred protection against intracellular pathogens and Th2 cells (humoral immunity or antibody-mediated protection) conferred immunity against extracellular pathogens was formulated (20). In view of recent knowledge, this view is limited, especially as it has been demonstrated that antibodies participate in all aspects of the immune response, from protecting the host during the initiation of infection to later challenge. Additionally the hallmark of the Th2 (humoral) response in mice and humans is the production of specific IgE (21). Therefore, the induction of IgE is not synonymous with humoral immune responses. Cellular immune responses were primarily considered as those inducing only cytotoxic T-lymphocytes (CTLs) and later on as Th1 cells (21), required to induce a good CTL with memory response. The Th1 immune response also induces an antibody (humoral) response. The main functional antibodies are IgG2a or IgG2c, depending on the mouse strain, or IgG1 and IgG3 in humans. Their biological function is determined by their capacity to fix complement (IgG2a or IgG2c) and by Fc receptors (IgG1). In humans, IgG1, the most presented and long-lasting isotype in blood (∼9 mg mL^−1^; half-life of 21 days) is the dominant isotype in a Th1 cytokine response. It has also been assumed that the Th2 or humoral immune response induces neutralizing antibodies, while the Th1 response induces opsonophagocytic and bactericidal effector functions. However, it is necessary to introduce a cautionary note since all human IgG subclasses, including IgA, induce a similar level of neutralization (22). In mice, the antibody isotypes that bind best to Fcγ receptors (such as IgG2a/2c) are also produced, in part, as a result of IFN-γ-mediated isotype switching of B cells (23). However, investigations have demonstrated that the production of antigen-specific IgE and specific IgG1 are not definitely correlated (24). The cytokine IL-4 appears not to be essential for IgG1 class switching, but plays a crucial role in IgE production (25). Consequently IgG1 in mice is not a predictor marker of Th2 immune response. Currently, the use of adjuvants has received much interest for allergen immunotherapy. The Th1-directing adjuvant, monophosphoryl lipid A (MPL^®^), is now in clinical use in allergy vaccines formulated with the depot adjuvant l-tyrosine (26). The clinical efficacy of an ultrashort course of ragweed pollen allergen adsorbed to l-tyrosine plus MPL^®^(Ragweed MATA MPL) in reducing allergy symptoms in patients with seasonal allergic rhinitis has recently been shown (27). In the field of anti-viral immunity, virus-like particles (VLPs) are considered a potent vaccine platform, proven to be immunogenic and clinically effective. In order to enhance immune cell activation, the addition of TLR ligands and/or depot-forming adjuvants seems to be useful for the treatment of allergic rhinitis (28). In a prophylactic approach, the grass pollen allergen Phl p 5 was administered by a skin patch with or without the Th1-promoting CpG oligodeoxynucleotide 1826 as an adjuvant. The results indicated that the addition of CpG balanced the response and prevented allergic sensitization, i.e., IgE induction, airway inflammation, and expression of T helper 2 cytokines (29).

### Secretory IgA antibody: An old friend and sentinel can be bolstered with mucosal adjuvants

In the pathogenesis of infectious and contagious diseases, over 90% of pathogens enter or are established at mucosal surfaces. The antibody isotype IgA is the main antibody that confers mucosal protection. IgA is considered a non-inflammatory effector (30, 31). However, it is not clear whether this IgA effector function is linked with a Th3 or a Th2 cellular pattern (32). Recent work has demonstrated that IgG is capable of mediating active humoral protection in several mucosal locations, but the kinetics of the response is totally different to that of IgA (33, 34). This is probably due to the mechanism of mucosal transportation; which is known to be passive for IgG (high blood concentration is required) and active for IgA. In addition, covalently conjugated polysaccharide vaccines, applied parenterally, contribute to the control of the infection by indirect protection by reducing the carriage rates (35) and herd immunity (36, 37), but salivary anticapsular IgA-levels seem to respond much better to natural boosting (38). Nevertheless, the influence of carriage over immunization is not clear. Another important set of observations is that not all locally induced antibodies are of the IgA isotype, in particular IgG has been found in several mucosal surfaces. For example, IgG concentrations exceed IgA in male and female genital tracts (39). This differential isotype distribution provides evidence for local immune origin, as distinct from the systemic compartment. One possible non-invasive method to detect mucosal, secretory IgA is through the analysis of saliva following salivary gland stimulation and IgA measurement. However, gut-associated and nasopharynx-associated lymphoid tissues do not contribute equally to the pool of memory/effector B cells that differentiate into mucosal plasma cells elsewhere in the body (40). Despite such problems, saliva remains an interesting biological fluid with great scientific and clinical potential (41). It has been recently stated that vaccine development initiatives should now focus on the development of mucosal vaccines, highlighting the need for the production of safe and potent adjuvants for mucosal delivery (42). At present, only a few attenuated oral whole-cell vaccines and nasal vaccines have been approved. Some of these have limited use (43). The rational design of mucosal adjuvants demands a better understating of the mucosal immune system and mechanisms governing its activation (44, 45). Consequently, several vaccine companies have been addressing these goals, but so far with limited or no success. Overall, more work is required to understand the mechanism of production of IgA and its role in mucosal protection. Searching for mucosal adjuvants could be an essential step in advancing the field of vaccinology.

### New studies of adjuvant-antigen vaccine formulations should accelerate the development of vaccines

In vaccine development, the choice of antigen is essential. However, vaccine formulations coupled to the correct adjuvants might be decisive in developing an effective vaccine formulation against life-threatening and neglected infectious diseases. Many recent vaccine projects have faced the problem of antigenic variation. The diversity of antigens has been difficult to classify. Some microorganisms, for example *Streptococcus pneumoniae and N. meningitidis*, exhibit little antigenic variability within a host, but show extensive population-wide variation that can change in a given demographical niche and time (46). Antigenic variation over time can be rapid, as with the influenza virus, where a new vaccine is required every year, and HIV and Hepatitis C virus, where spontaneous mutations during viral replication make it impossible to select a single protective antigen for use in a vaccine For vaccinologists, searching for stable or cross-reactive antigens, the biophysical characterization of antigens, assessing how antigens and adjuvants interact, and formulating stability should be the basis of a systematic approach to the development of effective, safe, and inexpensive vaccines. Currently, the most used adjuvant in licensed products is Alum, which acts as a DS. The critical aspect in this type of formulation is antigen adsorption. Alternatively, adjuvants can be a complex of multiple substances: MF59, an oil-in-water emulsion composed of small droplets of squalene surrounded by a monolayer of non-ionic detergents is an example. However, the stimulatory capacities of these adjuvants are only present when they are fully formulated (47). GlaxoSmithKline (GSK, Belgium) has developed several adjuvants systems (AS) that combine classical adjuvants with immunomodulators specifically adapted to the antigen and the target population (48). AS04 (a combination adjuvant composed of MPL A (a TLR-4 ligand) adsorbed to Alum) is licensed for use, in GSK’s Cervarix vaccine against human papilloma virus and the vaccine against hepatitis B virus (49). This formulation includes IP and DS properties. Currently, evidences in the work with AFCo1 and AFPL1 without Alum, with incorporated or co-administered antigens, have demonstrated that IP and IPz properties are contained in the same structure as endogenous adjuvants essential for their immunogenicity. Nevertheless, industry-quality adjuvant production and relevant antigen-adjuvant formulations should be considered as key factors by vaccine manufacturers and vaccine development programs. The following section looks at the Finlay adjuvants in more detail.

## Finlay Adjuvant Platform: A Different Approach from Existing Adjuvant Types

**Figure 1:**
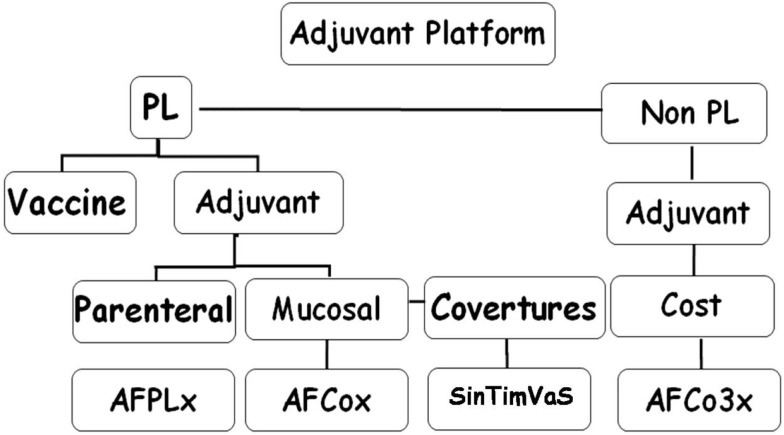
**Finlay adjuvant platform**. PL, proteoliposome, nanovesicles extracted from bacterial outer membrane (OMV); AFPLx, adjuvant Finlay PLx extracted from different bacteria; AFCo (Cochleate)x obtained from PLx; STVS, single-time vaccination strategy; AFCo3, Co obtained from non-derived-PL structure.

The Finlay Adjuvant (AF) platform consists of a series of proprietary adjuvants (50–55). They combine three constituents: (i) nanovesicles (PL) extracted from bacterial outer membranes that contain protective antigens (56–58), in addition to adjuvant components; (ii), PL-derived Co (microparticles), which conserve the protective and adjuvant PL components; and (iii), non-PL derived Co (microparticles), with MAMPs as the main component (Figure 1).

### Adjuvant Finlay proteoliposome in alum, increases its stability, reduces its pyrogenicity, and induces Th1 polarization

The adjuvant AFPL1 is a complex nano-structure consisting of vesicles extracted from the outer membrane of *N. meningitidis* serogroup B (B:4:P1,19,15:L3,7,9 strain). The vaccine properties of these outer membrane vesicles (OMVs) adsorbed onto alum gel was described and patented as part of the VA-MENGOC-BC^®^ vaccine (58, 59). The first demonstration that AFPL1 induces a preferential Th1 polarization in humans was described by Lapinet et al. (60) and Pérez et al. (61). The presence of IFNγ and IL-2 mRNAs was observed in peripheral blood mononuclear cells obtained from immunized subjects after *in vitro* challenge with AFPL1. AFPL1 also stimulated production of pro-inflammatory cytokines (TNF-α, IL-1β, and IL-8) and chemokines (MIP1-α and MIP1-β) by neutrophils. It was later demonstrated that AFPL1 stimulates specific CD4+ and CD8+ T cells and also elicits innate immunity activation inducing chemokines, pro-inflammatory cytokines, and co-stimulatory molecules. These products confirmed the adjuvant properties of the first AF described by Pérez et al. (62) and Pérez Martín et al. (51). AFPL1 can be adsorbed onto Alum; the degree of adjuvant adsorption is an important property that is related to the additional IP, DS, and IPz capacities of this preparation.

**Figure 2:**
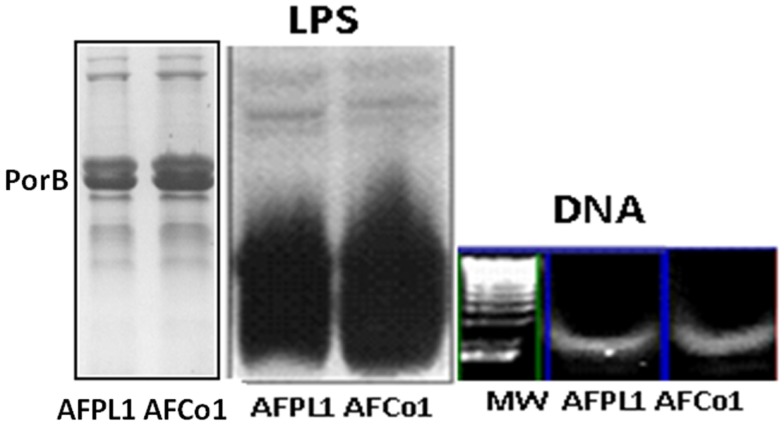
**Three main microbial-associated molecular patterns present in AFPL1 and AFCo1**. Lipopolysaccharide (LPS) Porin B (PorB), and traces of bacterial DNA detected by SDS-PAGE stained with Coomassie blue for PorB and silver staining for LPS and agarose gel for DNA.

AFPL1 contains native lipopolysaccharide (LPS), PorB, traces of bacterial DNA, three synergistic MAMPs that interact with TLR-4, TLR-2, and TLR-9, respectively, as immunopotentiator molecules (Figure 2), which can be considered as their own endogenous adjuvants that are essential for vaccine immunogenicity. The nano-particle structure of the adjuvant, with negative and positive lipids and several glycoproteins, can package heterologous proteins and can be readily used as a vaccine-DS capable of enhancing the immunogenicity of exogenous protein antigens. In addition, studies in our laboratory have shown that the IPz effect is not limited to LPS (63). This combination vaccine may explain the preferential Th1 immune response, cross-priming, and *in vivo* CTL response characterized by the production of IL-12 and IFNγ and delayed-type hypersensitivity. It might also explain the Th1 subclasses (IgG2a in BALB/c and IgG1 and IgG3 in human) and lack of IgE or IL-4/IL-5 production in mice and humans. Previous studies have shown that the OMV stimulate pro-inflammatory responses and induce immunoprotection against colonization or pathogenic challenge (64–66). For example, the Novartis MenB vaccine (4CMenB, Bexsero^®^), which received EU approval, includes three major antigens, identified by reverse vaccinology, and 25 μg of detoxified OMV from strain NZ 98/254 (67, 68). Two other vaccines contain similar PL: the meningococcal vaccine, which includes a PL component, and received EU approval (68), and the malaria vaccine RTS,S/ASO1, developed by GSK, which includes PL as the liposome component of the AS01 adjuvant (69).

Stability is one of the requirements for any pharmaceutical formulation as well as low or absence of pyrogenicity. Therefore, adjuvants that contain native LPS should be monitored carefully. The adjuvant AFPL1 has LPS inserted in the structure and is never free. In addition, the consistency of GMP PL production at the Finlay Institute and the number of doses (more than 55 × 10^6^) administered in adults and infants, as part of the National Immunization Program, guarantee that AFPL1 is considered a safe adjuvant *per se*. Further DS properties are introduced by the incorporation of AFPL1 onto alum gel. Importantly, this facilitates the commercialization process, since it increases safety and particle stability. In addition, it changes the suggested Th2 pattern of Alum to a Th1 pattern, increasing its stability for years, and reducing its pyrogenicity.

### The Finlay adjuvant cochleate 1 does not require aluminum hydroxide to work following parenteral and mucosal administration

**Figure 3:**
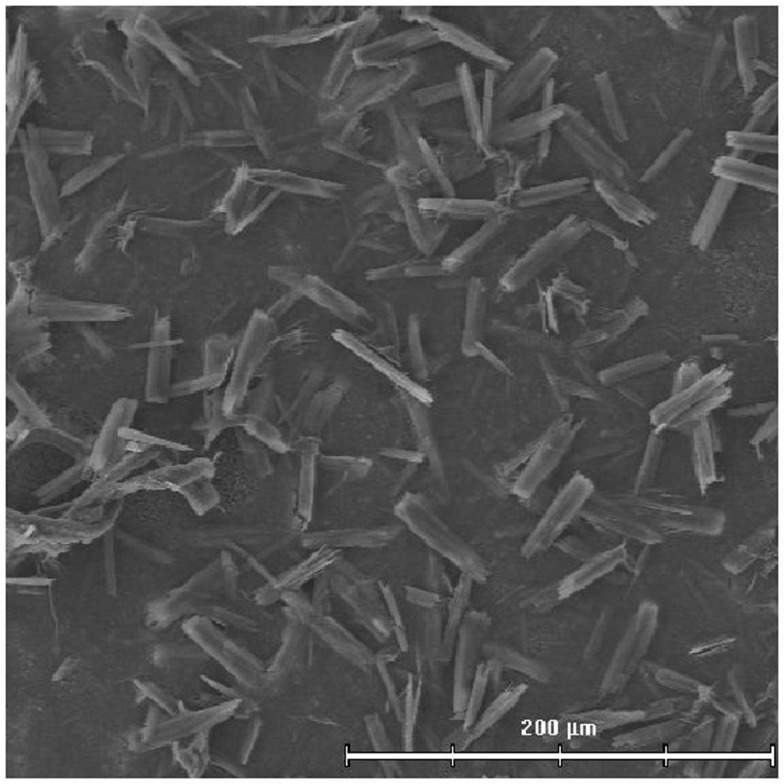
**Electron photograph of AFCo1 obtained by close cycle technology**.

AFCo1 is a PL-derived Co microparticle produced using different techniques (simple dialysis, rotary dialysis, or flow filtration) (70). Recently, in order to produce this adjuvant our laboratory has developed a closed cycle technology that is currently under expansion. Closed cycle technology permits the production of a sterile product. PL is dissolved in a buffer containing Tris 30 mmol/L and 1% (w/v) sodium deoxycholate, pH 7.4 to a final protein concentration of 1 mg/mL. Then, formation buffer (Tris 30 mmol/L, CaCl_2_ 10 mmol/L, and NaCl 100 mmol/L, pH 7.4) is added. Excess detergent and calcium is removed by centrifugation at 3000 × *g*, 10 min with Tris buffer (30 mmol/L, pH 7.2) and stored at 4°C. The Co formation is marked by the appearance of a white suspension. The efficiency of the process is estimated by protein quantities in the precipitate and the supernatant. The AFCo1 microparticles maintain the same IP, DS, and IPz properties of the AFPL1 precursor, and are stable. Alum is not required (Figures 2 and 3).

AFCo1 is an effective mucosal adjuvant in mice, when administered via different mucosal routes (nasal, oral, rectal, and vaginal) and combined with different antigens (ovalbumin, Ova; glycoprotein gD2 from Herpes Simplex Virus, HSV; bovine serum albumin, BSA; proteins, and peptides) (71, 72). AFCo1 administered by the nasal route induces systemic and mucosal (at local and distal sites) immune responses and total protection against HSV challenge (73).

**Figure 4:**
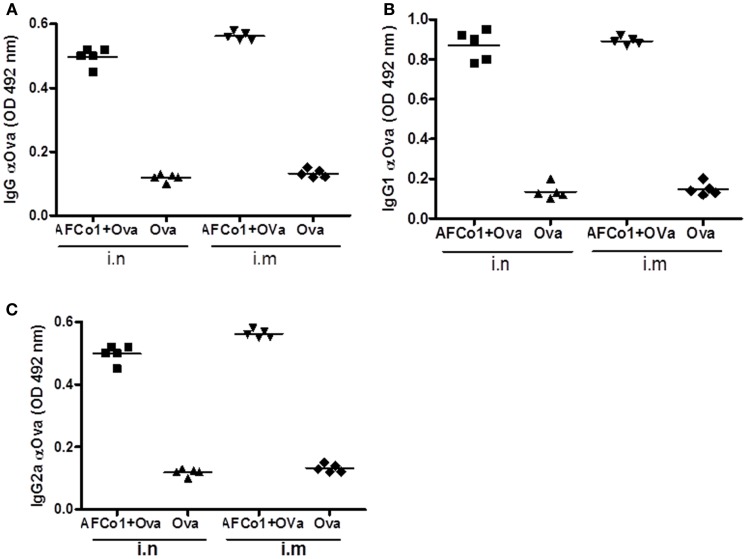
**Antibody response induced by AFCo1 plus Ovalbumin (Ova) by nasal (i.n) and intramuscular (i.m) routes**. **(A)** IgG; **(B)** IgG1; and **(C)**, IgG2a. BALB/c mice were distributed in two immunized groups and a control group. The first group was immunized with three i.n doses (0, 7, 14 days) of AFCo1 + Ova (25 μg/20 μg in 25 μL, 12.5 μL through each nostril). The second was treated with two i.m doses (0, 14 days) of AFCo1 + Ova (12.5 μg/10 μg in 50 μL *per* animal). The control received Ova i.n or i.m at 20 or 10 μg, respectively. Serum samples were taken 15 days after the last dose and antibody determination was carried out by ELISA. The figure shows the average and standard deviation of the mathematical relationship of values (OD) of two determinations in three independent experiments. The different *p* denote significant differences according to a Tukey multiple comparison test (*p* < 0.05).

AFCo1 is a microparticle with an approximate diameter of 11 μm. Although we predicted it would not work via the parenteral route, two intramuscular doses of AFCo1 plus Ova induced a similar systemic immune response of IgG, and subclass response (IgG1 and IgG2a), equivalent to three nasal doses in mice (Figure 4). This result suggests that the DS capacity of AFCo1-containing lipids and Ca^2+^ (known membrane perturbation and disruption agents) could take place through natural membrane fusion mechanisms, permitting interaction with several antigen-presenting cells without the requirement of internalization (74). Thus AFCo1 works via mucosal as well as parenteral routes. Currently, AFCo1 is in an advanced stage of clinical development with clinical trials on the horizon.

### Finlay adjuvant proteoliposomes adsorbed onto alum gel overcome the Th2 pattern induced by allergens

**Figure 5:**
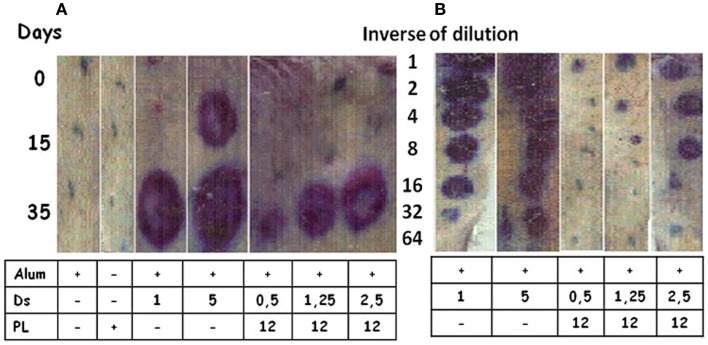
**Passive cutaneous anaphylaxis**. Male rats were inoculated subcutaneously with 100 μL of sera from mice immunized intramuscularly with two doses 15 days apart of *Dermatophagoides siboney* (Ds) allergen adsorbed onto aluminum hydroxide (Alum) at 1 or 5 μg *per* dose, or adjuvanted with AFPL1 (PL at 12 μg) in Alum at Ds concentrations of 0.5–2.5 μg *per* dose. Controls included AFPL1 or Alum alone. Sera were prepared at 0, 15, and 35 days. After 48 h, Evans Blue plus 1 mg of Ds was inoculated intravenously. Then the rats were sacrificed and the skin was used to evaluate the density and diameter of the stain. **(A)** Shows the evaluation of undiluted sera and **(B)** the quantity of the specific IgE dilution in the sera.

Allergens are inducers of a Th2 pattern, the hallmark of which is IgE production. *Dermatophagoides siboney* is a house dust mite, which in Cuba, is the main causative agent of allergy reactions. The Th2 response induced by Der s 1 and Der s 2 (the main *D. siboney* allergens) in Alum in unprimed mice was overcome by the formulation of AFPL1 adsorbed onto Alum. This induced IgG2a, IFNγ, and caused the reduction of specific and total IgE (50, 75). Specific IgE was measured by passive cutaneous anaphylaxis in male rats challenged with sera from immunized mice with 3 allergen concentrations (0.5; 1.25, or 2.5 μg/dose) and AFPL1 (12 μg/dose) in Alum. A reduction of the intensity of inflammation was 132, 42, and 37 times respectively (Figure 5A). When sera from immunized mice were diluted and retested, the positive dilutions observed were in the undiluted, 1:4, and 1:8 samples, respectively (Figure 5B). The efficiency of this formulation was also tested by the subcutaneous route in allergen unprimed and sensitized mice. This formulation has been concluded as satisfactory in preclinical toxicity and stability tests. A Phase I clinical trial using only three doses was approved by CECMED, the Cuban regulatory agency, and is in progress (DA-EC2012014). Overall, AFPL1 changes the Th2 behavior of *D. siboney* allergen in unprimed and sensitized mice and is a promising human vaccine.

### Single-time vaccination strategy to increase coverage using Finlay adjuvants

Few vaccines are administered as a single dose. Also, it has been difficult to obtain a good immune response using non-living antigen vaccines as a single dose. With such vaccines, multiple doses are generally needed to provide sufficient stimulation of the immune system, and to achieve durable responses over time. Thus, a complete immunization schedule is mandatory for protection. The successful delivery of active vaccines depends amongst other factors on effective vaccine storage and distribution, including cold chain management. The cold chain aspect alone can account for 80% of the financial cost of a given vaccination program (76). The cost of the vaccination program is high in low-resource regions with poor vaccine affordability. Therefore, the development of new immunization strategies and procuring a single-dose vaccine are of pivotal importance.

**Figure 6:**
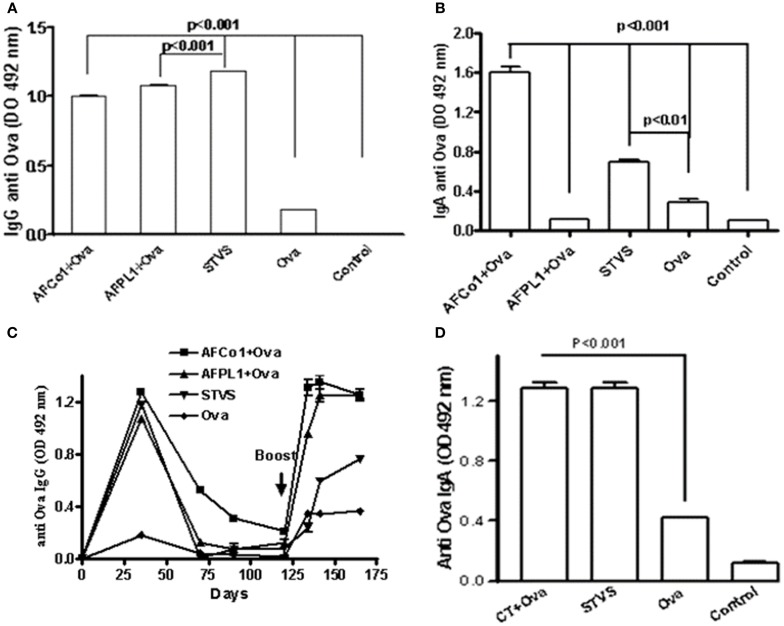
**Specific anti-Ova immune response induced by single-time vaccination strategy (SinTimVaS)**. BALB/c mice were immunized with: three i.n. doses (0, 7, 14 days) of AFCo1 + Ova (50 μg/25 μg in 25 μL per animal, 12.5 μL through each nostril); two i.m. doses (0, 14 days) of AFPL1 + Ova (12.5 μg/10 μg in 50 μL *per* animal); both treatments at the same time in SinTimVaS and Ova as the control. Other groups were followed until specific IgG decreased and a booster Ova dose at day 125 was administered. Other groups using cholera toxin (CT) as the adjuvant instead of AFCo1/AFPL1 were also evaluated. These were administered via the nasal route CT + Ova (5 μg/50 μg in 25 μL *per* animal, 12.5 μL through each nostril) and simultaneously one intramuscular dose of CT + Ova (5 μg/20 μg in 50 μL) was administered. For anti-Ova IgG or IgA, serum samples at 21 days after the last dose were used. The determination was carried out by ELISA. Data were expressed as averages and standard deviation of OD of two determinations in three independent experiments. Specific Ova IgG **(A)**; **(B,D)** specific Ova IgA; and **(C)** specific Ova IgG after a booster Ova dose. Significant differences between the means of different groups were determined by a Tukey multiple comparison test using Graph Pad Prism 4 software (Calif.). A *p*-value of <0.05 was considered statistically significant.

Many studies combine nasal and/or oral routes with intramuscular immunizations, using the mucosal route as a priming dose and the parenteral route for the booster dose (traditional prime boosts spaced doses) or *vice versa* (77, 78). We have developed a novel immunization approach called single-time vaccination strategy (SinTimVaS) (79). In this approach a combination of two priming doses (one mucosal and one parenteral) is given simultaneously by different routes without the requirement for a subsequent boost. The use of potent adjuvants is a key factor in this novel strategy. González et al. (79) demonstrated that simultaneous vaccination such as intramuscular administration of AFPL1 and nasal administration of AFCo1 induced systemic and mucosal responses against *N. meningitidis* serogroup B. We have recently shown that SinTimVaS applications of tetanus toxoid or BSA combined with AF induced antigen-specific mucosal and systemic responses. Similar results were obtained using Ova as a weaker model antigen. This induced a similar systemic anti-Ova IgG response to two parenteral doses of AFPL1 plus Ova and three nasal doses of AFCo1 plus Ova (Figure 6A). Only AFCo1 plus Ova and SinTimVaS induced anti-Ova IgA (Figure 6B) and a memory response (Figure 6C). We also found that our immunization strategy not only works with AF, but also when cholera toxin is used as a mucosal adjuvant (Figure 6D). The strength of this strategy is that it can achieve high vaccine coverage, reducing the logistics and the number of follow-up and catch-up campaigns. It also induces similar systemic and mucosal immune responses. However, further studies using animal models as well as humans are needed to explain the immune mechanisms involved in this strategy.

### Finlay adjuvants change the T-independence pattern of non-covalently conjugated polysaccharides

Encapsulated bacteria have an outer covering composed of capsular polysaccharides (Ps). The Ps are T-independent type 2 antigens (TI-2), based on their ability to stimulate antibody production in the absence of T-cell help (80). Thus, vaccination with TI-2 antigens elicits primarily IgM with limited class switching, affinity maturation, and immunological memory (81). Vaccines composed of Ps are immunogenic, provide protection in healthy adults and reduce the risk of invasive disease (82), but have low immunogenicity in children younger than 2 years of age (83). Also, these vaccines have demonstrated lack of booster responses to plain Ps challenge and an absence of affinity maturation of Ps specific antibodies (84). When Ps is covalently conjugated to a carrier protein, conferring the immunological attributes of the carrier on the attached Ps, it elicits T-cell help for B cells, inducing large-scale IgM to IgG switching of B cells to long-lived plasma cells or memory B cell development (85). Vaccination with conjugate vaccines increase the amount of specific IgG antibodies produced and increases the IgG:IgM ratio on repeated vaccination. The IPz effect of Finlay adjuvants has been tested against multiple protein antigens such as: merozoite surface proteins from *Plasmodium falciparum* (86), synthetic peptides and recombinant proteins from *Streptococcus pyogenes* (87). However, the incorporation of these adjuvants that possess the ability to trigger multiple TLR agonists, into plain Ps formulations could effectively avert the TI-2 of Ps. We have demonstrated that nasal immunization of AFCo1 plus Ps from *N. meningitidis* serogroup C induces PsC-specific mucosal and systemic immune responses (88).

**Figure 7:**
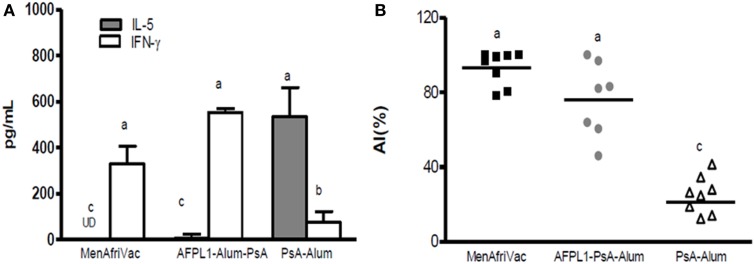
**Th1 cytokine profile and antibody affinity maturation after a plain PsA booster**. Groups of mice received 5 μg PsA adsorbed onto Alum or together with 5 μg of AFPL1 or conjugate vaccine MenAfriVac^®^ via the subcutaneous route. Immunizations were performed twice, 21 days apart. A subcutaneous booster of plain PsA was performed 105 days after the last dose. Spleens from individual mice after the booster immunization were pooled within each group. **(A)** Cytokine levels, measured by ELISA are expressed as pg/mL ± SEM. **(B)** affinity index (AI) was determined by avidity ELISA with 250 mM sodium thiocyanate. The AI, percentage of antibodies that remain bound to the antigen after treatment with the chaotropic agent, was calculated using the following formula: AI = titer (NaSCN+)/titer (NaSCN−). Results are expressed as the geometric mean of the affinity index (%). Data were analyzed by one-way ANOVA followed by a Tukey’s multiple comparison test. A *p*-value of <0.05 was considered statistically significant and is represented by different letters.

Furthermore, studies with the Cuban bivalent vaccine (VA-MENGOC-BC^®^), which contains non-covalently conjugated PsC, showed that teenagers vaccinated in their infant life induced a significantly specific serogroup C response after a third dose of VA-MENGOC-BC^®^ or to natural *Neisseria* challenge (89). We have also shown that subcutaneous immunization of AFPL1 plus Ps from *N. meningitidis* serogroup A (PsA) induced increased antibody affinity and a Th1 cytokine pattern after plain PsA booster (Figures 7A,B). In addition, Romeu et al. (90) demonstrated that a combined formulation of PLs from meningococcal serogroups A and W can stimulate cellular immunity and long-term memory cells against PsA, increasing affinity maturation after a plain Ps booster (90). The presence of several synergistic TLR agonists in the structure of these adjuvants influences anti-Ps antibody production, T-cell help, activation, and the proliferation of memory cells, opening new perspectives in the application of plain Ps antigens without the requirement of covalent conjugation. Therefore, the combination of Ps (conjugated or not) and Finlay adjuvants has the advantage of changing the TI-2 pattern of capsular Ps, which could have important implications for vaccinology. The mechanism of Ps presentation to T-cells described recently for Ps covalently conjugated vaccines (91) could possibly be extended to Ps-non-covalently conjugated formulations.

### The application of AFCo3 as an immunopotentiator in aquaculture

**Figure 8:**
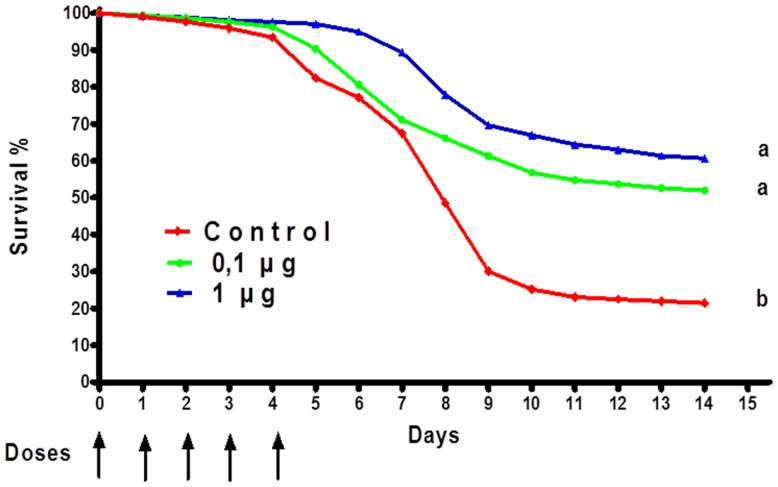
**Percentage of survival of *Clarias gariepinus* immunopotentiated with AFCo3a**. Oral treatments of AFCo3a mixed with the first daily food for five consecutive days. Survival was determined every day for 15 days. Statistical differences were determined by a Kruskal–Wallis/Mann–Whitney *U*-test. A *p*-value of <0.05 was considered statistically significant and is represented by different letters.

Aquaculture is one of the fastest growing economic activities in food production (92). One of the main challenges is to obtain a high-volume production of fish with the highest possible quality (93). Teleost fish, which mainly secrete IgM, display a strong innate immune response (94), but their adaptive immune response is relatively weak. In aquaculture, adjuvants and IPs in aquaculture have been used to improve the innate defense of fish and to promote healthy growth (95). The use of IPs constitutes a viable strategy to reduce losses from health problems in the aquaculture sector (96). Since the discovery of TLRs in fish, they have become of special interest in understanding host-pathogen interactions. LPS is known as one of the most potent IP in mammals. However, in fish, LPS is considered to have a low pro-inflammatory potential, probably due to the fact that the TLR-4 genes found in similar aquatic organisms such as zebrafish do not recognize the mammalian agonist (97). However, cells and immune system components, both systemic and at the surface (intestinal and gill), are activated and mobilized in response to LPS (98). For example, immunization of the common carp (*Cyprinus carpio*) with LPS from *Aeromonas hydrophila* yielded an improved immunity and better survival (99). Grass carp (*Ctenopharyngodon idella*) injected intraperitoneally with LPS, outer membrane proteins or formalin killed cells from *A. hydrophila* induced a relative percent survival of 83.3, 72.2, and 55.6%, respectively. This suggests that LPS and outer membrane proteins could be important in the development of vaccines against *A. hydrophila* in grass carp and other fish (100). Studies conducted by Nayak et al. (101) using the Indian major carp showed that LPS from three different gram-negative bacteria have IP potency (101). We have been working on the development of a novel microparticle adjuvant series called AFCo3a, which contains LPS isolated from meningococcal bacteria (54). We are currently testing this preparation using the African catfish, *Clarias gariepinus*. Oral treatment of AFCo3a mixed with the first daily food administered for five consecutive days induced an improved survival rate compared with a control group (Figure 8). We also detected a significant (*p* < 0.05) increase in the production of IgM in fish treated orally with formalin-inactivated *A. hydrophila*, adjuvanted with AFCo3a. Our data suggests that oral application of potent IP-like Finlay adjuvants in aquaculture can be an effective and economical way to treat fish under intensive culture conditions. This is pertinent especially in view of the fact that the main losses in this industry are caused by excessive handling and high-density fish production.

## Summary and Final Remarks

With the increasing desire to obtain novel effective adjuvants to future vaccines this field is experiencing a renaissance. This paper illustrates the current state of affairs regarding the influence of existing adjuvants and terminologies that can properly guide adjuvant-vaccine research and formulation. It also summarizes how adjuvants are key factors for the development of future vaccines and the lessons learned from the Finlay Adjuvant platform. The key advantage of AFPL1 is its safety in humans, as a component of the prophylactic meningococcal vaccine. The recent development of the AFPL1 adjuvant as an allergen therapeutic vaccine has demonstrated effectiveness in reducing specific IgE and inducing preferential Th1 immune responses. Also, a new technology to produce PL or non-PL derived Co in a close cycle was developed, ensuring the production of sterile and scale-up products applicable not only to humans, but also in the veterinary field. The mucosal effectiveness of Co, their use in SinTimVaS permits increasing vaccination coverage and reducing campaign costs. The IPz properties of Co and PL to activate the innate immune response of fish and invertebrates could be a new field of application. The potential of the Finlay Adjuvants Platform to shape desired immune responses by stimulating multiple TLRs potentiating antigen-induced responses and providing not only prophylactic, but also therapeutic protection against infectious and non-infectious diseases was highlighted.

A better understanding of the mechanism of activation of the mucosal immune system, homing to effector sites and the role of mucosal IgA in mucosal protection is essential to provide mucosal protection against pathogens. More work should be addressed to find simple and affordable methods for the evaluation of cellular responses in the vaccination field, in so doing, cellular and mucosal vaccine protection correlates can be established. The applicability of AFPL1 as a nano-particle adjuvant to other vaccines and the clinical introduction of AFCo1, preferably with mucosal vaccine candidates or in SinTimVaS should be an essential step to consider. In addition, information gained in studies developed with non-conjugated-polysaccharides, provides opportunity to take rational approaches in the design of formulations capable of overcoming the T-independence of polysaccharides.

## Conflict of Interest Statement

The authors declare that the research was conducted in the absence of any commercial or financial relationships that could be construed as a potential conflict of interest.
